# Design and Optimization of Self-Nanoemulsifying Drug Delivery Systems to Enhance the Anti-Inflammatory Efficacy of *Derris scandens* (Roxb.) Benth. Extract

**DOI:** 10.3390/pharmaceutics18091188

**Published:** 2026-09-20

**Authors:** Nattakanwadee Khumpirapang, Kantapich Srikham, Thapakorn Somboon, Krit Suknuntha, Daniela Gruľová, Neti Waranuch, Kornkanok Ingkaninan, Siriporn Okonogi

**Affiliations:** 1 Department of Pharmaceutical Chemistry and Pharmacognosy, Faculty of Pharmaceutical Sciences, Naresuan University, Phitsanulok 65000, Thailand; nattakanwadeek@nu.ac.th; 2 Cosmetics and Natural Products Research Center, Faculty of Pharmaceutical Sciences, Naresuan University, Phitsanulok 65000, Thailand; kantapich.sr@nu.ac.th (K.S.); netiw@nu.ac.th (N.W.); 3 Center of Excellence for Innovation in Chemistry, Faculty of Pharmaceutical Sciences, Naresuan University, Phitsanulok 65000, Thailand; thapakorn.so@nu.ac.th; 4 Department of Pharmaceutical Chemistry, Faculty of Pharmaceutical Sciences, Prince of Songkla University, Songkhla 90110, Thailand; krit@pharmacy.psu.ac.th; 5 Department of Ecology, Faculty of Humanities and Natural Sciences, University of Prešov, 08001 Prešov, Slovakia; daniela.grulova@unipo.sk; 6 Department of Pharmaceutical Technology, Faculty of Pharmaceutical Sciences, Naresuan University, Phitsanulok 65000, Thailand; 7 Center of Excellence in Pharmaceutical Nanotechnology, Faculty of Pharmacy, Chiang Mai University, Chiang Mai 50200, Thailand

**Keywords:** anti-inflammatory activity, *Derris scandens*, design of experiment, lupalbigenin, nanodelivery system, nanoemulsion, topical formulation

## Abstract

**Background/Objectives:** *Derris scandens* (Roxb.) Benth. stem extract (DSE) is used in Thailand for musculoskeletal pain, but its bioactive, lupalbigenin, has limited aqueous solubility. This study aimed to develop a DSE-loaded self-nanoemulsifying drug delivery system (DSE-SNEDDS), characterized its nanoemulsions, and compared their anti-inflammatory activity and stability. **Methods:** Various extraction methods were compared to obtain a high yield of lupalbigenin. A D-optimal design was used to optimize SNEDDS composition based on the apparent solubility of lupalbigenin. The selected preconcentrate was diluted fivefold to prepare DSE nanoemulsion (DSE-NE). Droplet characteristics, RAW 264.7 cell viability, LPS-induced nitric oxide production, and 12-week stability at 4, 25, and 40 °C were assessed. **Results:** Ultrasound-assisted extraction gave the highest lupalbigenin content (108.69 ± 0.12 µg/mg DSE). The selected formulation contained 20% Lexol^®^ GT-865, 40% Kolliphor RH40, 30% Transcutol, and 10% ethanol. Fivefold dilution produced DSE-NE with a droplet size of 172.5 ± 0.3 nm and polydispersity index of 0.31 ± 0.01. Its IC_50_ was 4735 µg/mL formulation (94.70 µg/mL DSE equivalent), compared with 31.57 µg/mL for DSE in DMSO. At 40 µg/mL DSE equivalent, DSE-NE and DSE in DMSO inhibited nitric oxide production by 78.79 ± 2.31% and 50.78 ± 2.95%, respectively; matched blank NE produced 36.53 ± 6.45% inhibition. During storage, the preconcentrate retained 95–96% of lupalbigenin and generated nanoemulsions with minimal size changes, whereas preformed DSE-NE retained 81–87% and showed greater droplet growth. **Conclusions:** These results support the selection of the preconcentrate as the preferred storage form for further topical formulation development. However, skin delivery, dermal safety, and topical efficacy remain to be established.

## 1. Introduction

Musculoskeletal disorders are a major cause of pain, disability, and healthcare expenditure worldwide [1]. Muscle pain is one of the important symptoms of musculoskeletal diseases and impacts quality of life in patients [2]. Non-steroidal anti-inflammatory drugs (NSAIDs) are frequently used to manage musculoskeletal pain and inflammation through inhibition of prostaglandin synthesis [3]. Despite their effectiveness, prolonged or inappropriate use may be limited by gastrointestinal, renal, cardiovascular, and hypersensitivity-related adverse effects. These limitations have encouraged continued investigation of medicinal plants with established traditional uses in pain and inflammatory conditions.

**Figure 1:**
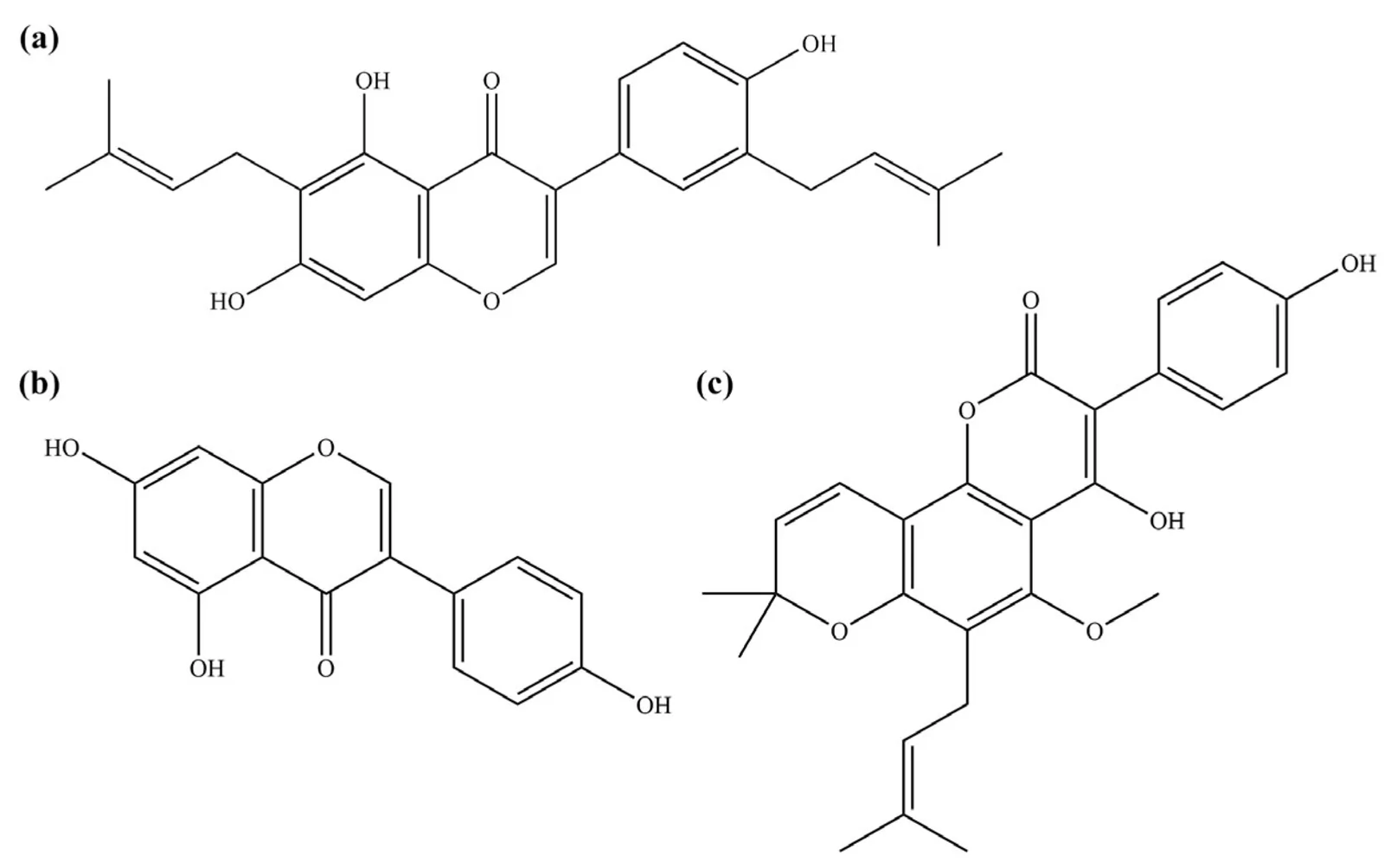
Chemical structures of the major bioactive constituents of *D. scandens*: lupalbigenin (**a**), genistein (**b**), and scandenin (**c**).

In recent years, researchers have increasingly explored medicinal plants as potential alternatives to conventional drugs. *Derris scandens* (Roxb.) Benth. is a woody climbing plant belonging to Fabaceae, also known as Leguminosae. This large plant family comprises six subfamilies and includes herbs, shrubs, trees, and climbing species [4]. Fabaceae plants produce diverse secondary metabolites, particularly flavonoids and isoflavonoids, many of which have biological and medicinal activities [5]. In Thai traditional medicine, the stem of *D. scandens* is used to relieve muscle, bone, and joint pain and to manage symptoms associated with osteoarthritis [6]. Evidence from clinical studies has supported its use for musculoskeletal pain [7], and both powdered stem and ethanolic stem extract are included in the Thailand National List of Essential Medicines [8]. The principal constituents associated with its anti-inflammatory activity include lupalbigenin, genistein, and scandenin (Figure 1). Their concentrations, particularly that of lupalbigenin, may vary according to the extraction conditions; therefore, the extraction procedure is an important consideration when controlling DSE quality. Gastrointestinal adverse events have also been reported following oral administration of *D. scandens* capsule [9].

The localized use of *D. scandens* for musculoskeletal pain provides a rationale for considering its subsequent development as a cutaneous formulation. In this context, dermal delivery aims to retain or deliver active compounds locally within the skin and underlying tissues, whereas transdermal delivery is intended to transport compounds across the skin into the systemic circulation. Both approaches differ from oral administration and must address the barrier properties of the stratum corneum [10,11]. Microemulsion-based systems have been investigated as one approach for improving the cutaneous delivery of poorly soluble compounds [12]. Nevertheless, the suitability of DSE for dermal application cannot be assumed from its traditional or oral use. Skin permeation, local deposition, irritation, and efficacy must be investigated before any benefit of cutaneous delivery can be established.

Nanotechnology-based formulations have been widely studied as a means of improving the solubilization and stability of poorly water-soluble compounds and, in some cases, enhancing their bioavailability [13,14,15]. Commonly investigated nanocarriers include drug nanocrystals, polymeric micelles, and lipid-based drug delivery systems (LBDDS) [16]. LBDDS were considered suitable for DSE because they can incorporate lipophilic constituents into a liquid formulation and can generally be prepared using relatively straightforward manufacturing processes. Their pharmaceutical applications, production considerations, and potential for further development have been extensively described [17,18,19]. This group includes self-microemulsifying drug delivery systems, microemulsions, liposomes, self-nanoemulsifying drug delivery systems (SNEDDS), and nanoemulsions (NE).

SNEDDS are anhydrous, isotropic preconcentrates composed of oil and surfactant, usually in combination with a co-surfactant or co-solvent [20]. When diluted with water under mild agitation, these systems form fine oil-in-water dispersions. Most reported applications of SNEDDS involve oral administration, where gastrointestinal fluids provide sufficient water for dispersion. In oral SNEDDS, absorption-enhancing excipients and surfactants such as Cremophor have been investigated for their effects on drug dissolution and intestinal absorption [21]. The conditions associated with cutaneous administration are different because only a limited amount of water is available on the skin surface. Therefore, a SNEDDS preconcentrate cannot be assumed to undergo complete self-emulsification when applied directly to the skin. Moreover, the development of a dermal dosage form requires route-specific evaluation, including pH, rheological behavior, drug release, skin permeation and deposition, and local compatibility. For these reasons, DSE-SNEDDS was used in the present study as a preparation and storage form, whereas a preformed DSE-NE prepared by controlled aqueous dilution was used for biological evaluation.

The limited aqueous solubility of lupalbigenin, genistein, and scandenin, which are constituents of DSE associated with anti-inflammatory activity, provided the formulation rationale for this study. Genistein is a lipophilic compound with a log P of 3.04 and a molecular weight of 270.24 g/mol [22]. Scandenin and lupalbigenin are also lipophilic, with reported log P values of 5.4 and 6.5, respectively. These properties led us to investigate SNEDDS as a means of incorporating DSE into an aqueous dispersed system. Extraction methods were initially compared, after which a D-optimal design was applied to optimize DSE-SNEDDS using the apparent solubility of lupalbigenin from DSE as the formulation response. The selected preconcentrate was diluted under controlled conditions to prepare DSE-NE for droplet characterization and biological testing. The novelty of this work is the combination of DoE-based formulation optimization with a direct comparison between storage as the DSE-SNEDDS preconcentrate and storage as the preformed DSE-NE. A corresponding blank NE was also included to examine the contribution of the formulation excipients to the biological response. Furthermore, the physical and chemical stability of the DSE-SNEDDS preconcentrate and preformed DSE-NE were compared, and the in vitro anti-inflammatory activity of DSE-NE was evaluated.

## 2. Materials and Methods

### 2.1. Materials

**Figure 2:**
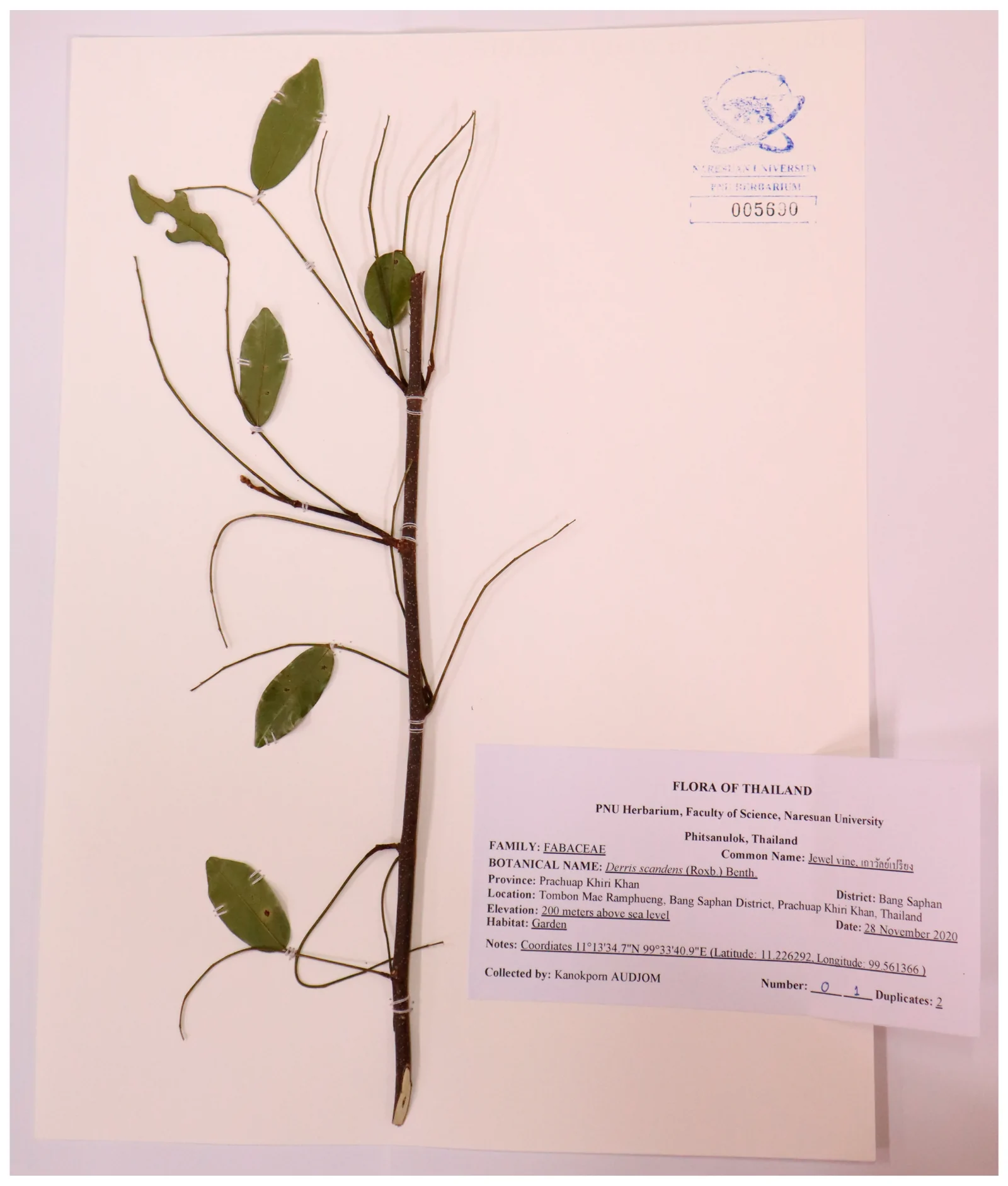
Herbarium voucher specimen of *D. scandens* (Roxb.) Benth.

Fresh stems of *D. scandens* were collected in November 2020 from a garden in Prachuap Khiri Khan, Thailand [Coordiates 11°13′34.7″ N 99°33′40.9″ E (Latitude: 11.226292, Longitude: 99.561366)]. Plant collection and handling followed the World Health Organization guidelines for good agricultural and collection practices for medicinal plants. The voucher specimen (PNU No. 005690) was prepared and deposited at the PNU Herbarium, Faculty of Science, Naresuan University, Thailand. The botanical identity of the collected material was confirmed by Assistant Professor Dr. Pranee Nangngam, Department of Biology, Faculty of Science, Naresuan University, Thailand. The deposited voucher specimen is shown in Figure 2.

Lupalbigenin was generously provided by Professor Dr. Apichart Suksamrarn from Faculty of Science, Ramkhamhaeng University, Thailand. RAW 264.7 macrophage cells were purchased from ATCC (Manassas, VA, USA). Fetal bovine serum (FBS), penicillin, streptomycin, Dulbecco’s Modified Eagle Medium (DMEM), trypsin-EDTA, and phosphate-buffered saline were purchased from Gibco (Life Technologies Corporation, Grand Island, NY, USA). Dimethyl sulfoxide (DMSO), 3-(4,5-Dimethylthiazol-2-yl)-2,5-diphenyltetrazolium bromide (MTT), *N*-1-napthylethylenediamine dihydrochloride, sulfanilamide, dexamethasone (DXA), and lipopolysaccharide (LPS) were from Sigma-Aldrich (St. Louis, MO, USA). A mixture of long-chain mono-, di-, and triglycerides (Maisine 35-1), caprylic/capric triglyceride (medium-chain triglyceride; MCT or Lexol^®^ GT-865), miglyol 812N, polyoxyl 40 hydrogenayed castor oil (Kolliphor RH40), and formic acid were purchased from Gattefossé (Saint Priest, France), INOLEX Group (Philadelphia, PA, USA), Cremer Oleo GmbH & Co. KG (Hamburg, Germany), BASF (Ludwigshafen, Germany), and QRec, (Auckland, New Zealand), respectively. Polyoxyethylene (80) sorbitan monooleate (Tween 80), polyoxyethylene (20) sorbitan monolaurate (Tween 20), ethanol (EtOH), di(ethylene glycol) ethyl ether (Transcutol), propylene glycol, and phosphoric acid were of analytical grade and supplied by Merck KGaA (Darmstadt, Germany). Furthermore, acetonitrile and methanol (HPLC grade) were purchased from RCI Labscan, Bangkok, Thailand.

### 2.2. Plant Extraction

The harvested stems of *D. scandens* were dried in a circulating hot-air oven at 45–50 °C until completely desiccated and then ground into a fine powder. The dried powder was subjected to extraction using three different methods, with a plant-to-solvent ratio of 1:10: (1) maceration in 95% EtOH for 24 h; (2) maceration in 95% EtOH for 24 h with shaking at 200 rpm; and (3) ultrasound-assisted extraction (37 kHz, 30 min) repeated three times at 25 ± 5 °C. The resulting extracts were filtered, and the filtrate was evaporated under reduced pressure until dry. The extracts were then analyzed for their isoflavone content using high-performance liquid chromatography (HPLC), and the extraction yields were recorded. All experiments were performed in triplicate.

### 2.3. HPLC Analysis

The isoflavone content of DSE (lupalbigenin) was analyzed using HPLC, with the analytical protocol modified and optimized based on previous studies [23,24]. The obtained extracts were dissolved in methanol, then filtered through a 0.22 µm nylon syringe filter, and then injected into the HPLC system. Quantitative analysis of lupalbigenin was conducted on a Shimadzu Nexera LC-40 (Shimadzu, Kyoto, Japan), equipped with a photodiode array detector (PDA). A Phenomenex Kinetex C18 reversed-phase analytical column (150 × 4.6 mm, 2.6 µm particle size; Phenomenex, Torrance, CA, USA) connected to a C18 guard column was used for separation. The mobile phase consisted of a mixture of 0.1% formic acid in acetonitrile (A) and 0.1% formic acid in water (B), running at a flow rate of 0.5 mL/min at room temperature (25 °C). The gradient elution program was as follows: 0–3 min, 75:25 (A:B, *v*/*v*); 3–13 min, 95:5; and 13–18 min, 75:25. A 5 µL injection volume of each sample was automatically injected into the analytical column, and the column effluent was monitored by the PDA detector. Standard curves for lupalbigenin were prepared for quantification. The samples were dissolved in methanol and filtered through a 0.22 µm nylon syringe filter (CNW Technologies GmbH, Shanghai, China) before injection. The amount of lupalbigenin was calculated from the peak areas using a linear standard curve with a concentration range of 2 to 100 µg/mL. The performance of the HPLC method was evaluated following guidance issued by the International Council for Harmonisation of Technical Requirements for Pharmaceuticals for Human Use. The validation experiments covered selectivity, linear response, limits of detection and quantification, precision, and accuracy. All experiments were performed in triplicate.

### 2.4. Preparation of DSE-SNEDDS

#### 2.4.1. Apparent Solubility of Lupalbigenin from DSE in Individual Excipients

An excipient-screening experiment was carried out to identify suitable components for the SNEDDS formulations. An excess of DSE was combined with 100 mg of each test excipient in separate 1.5 mL microcentrifuge tubes. The excipients examined were Miglyol 812N, Lexol^®^ GT-865 MB, Maisine 35-1, Kolliphor RH40, Tween 80, Tween 20, absolute ethanol, Transcutol, and propylene glycol. Each sample was mixed by vortexing and then maintained at 25 °C for 24 h to allow equilibration. After this period, the samples were centrifuged at 9170× *g* for 15 min using a Kubota 5922 refrigerated centrifuge (Kubota, Tokyo, Japan). A 10 µL portion of the resulting supernatant was diluted with 990 µL of absolute ethanol, corresponding to a 100-fold dilution, and passed through a 0.22 µm membrane filter. Lupalbigenin in the filtered samples was quantified using the HPLC procedure described in Section 2.3. The measured concentration was corrected for the dilution factor and reported as milligrams of lupalbigenin per gram of excipient. Because the experiment was performed using DSE rather than purified lupalbigenin, the calculated values represent the apparent solubility of lupalbigenin in the presence of other extract constituents. Excipients providing higher apparent lupalbigenin solubility were considered for subsequent formulation development.

#### 2.4.2. Experimental Design and Preparation of SNEDDS Formulations

The composition of DSE-SNEDDS was investigated using a D-optimal experimental design generated with MODDE version 13 (trial version; Umetrics, Umeå, Sweden). Two design matrices were constructed, one for the Tween 80 system and the other for the Kolliphor RH40 system. Each matrix consisted of 12 experimental runs, including three center-point replicates. The formulation variables were medium-chain triglyceride (MCT; (*X*_1_), 20–40% *w*/*w*), surfactant ((*X*_2_), 20–40% *w*/*w*), co-surfactant ((*X*_3_), 20–30% *w*/*w*), and co-solvent ((*X*_4_), fixed at 10% *w*/*w*), with the total composition constrained to 100% *w*/*w*. The response variable was the apparent solubility of lupalbigenin from DSE in each SNEDDS composition. Because the formulations contained the whole extract, this response reflects the behavior of lupalbigenin in the presence of other extract constituents and does not represent the intrinsic solubility of purified lupalbigenin. Quadratic models were fitted by partial least-squares regression. Model fit was described by goodness of fit (R^2^), while prediction power (Q^2^) was used to assess predictive performance; a (Q^2^) value above 0.5 was considered indicative of useful predictive ability [25]. Six additional formulations that were not included in model fitting (ET1–ET3 for the Tween 80 system and EC1–EC3 for the Kolliphor RH40 system) were prepared to compare the predicted and experimentally measured responses.

For each experimental run, the component quantities specified by the design matrix were weighed into a glass vial and mixed using a vortex mixer for 10 min. The resulting mixtures were stirred at 100 rpm for 24 h at room temperature and subsequently maintained at 37 °C for an additional 48 h [26]. Each formulation was prepared and analyzed in triplicate. The final formulation was selected by considering several predefined criteria together: Grade A self-emulsification, absence of phase separation after 24 h, a mean droplet size below 200 nm following aqueous dispersion, sufficient DSE loading as indicated by the apparent solubility of lupalbigenin, and the use of a simple composition containing pharmaceutically acceptable excipients. Apparent lupalbigenin solubility was therefore not used as the sole criterion for formulation selection.

#### 2.4.3. Investigation of Self-Emulsification Efficiency

Each SNEDDS formulation (100 mg) was added to 10 g of Milli-Q water at a SNEDDS-to-water mass ratio of 1:100. The water was obtained from an Advantage A10 purification system (Millipore, Billerica, MA, USA). The mixture was stirred at 100 rpm at room temperature, and the time required for emulsification together with the appearance of the resulting dispersion was recorded. Self-emulsification performance was classified into five grades according to the following criteria [27]:

**Grade A:** A transparent or slightly bluish nanoemulsion formed within 1 min.

**Grade B:** Emulsification occurred rapidly, producing a slightly less transparent dispersion with a bluish-white appearance.

**Grade C:** A fine and uniformly milky emulsion developed within 2 min.

**Grade D:** Emulsification required more than 2 min, and the resulting dispersion appeared dull grayish-white with slight oiliness.

**Grade E:** The formulation showed little or no self-emulsification, with large oil droplets remaining on the water surface.

### 2.5. Characterization of SNEDDS Formulation

The droplet size and size distribution (expressed as polydispersity index, PdI) of SNEDDS were characterized after dispersion those SNEDDS in Milli-Q water using photon correlation spectroscopy (PCS, Zetasizer Nano ZS, Malvern Instruments Ltd., Malvern, UK). The effect of volume of dilution media on SNEDDS droplet size and size distribution was studied. Three dilution folds (1:50, 1:100, and 1:1000 *w*/*w*) were compared. The size measurements were carried out at a fixed angle of 173° at 25 °C and the experiments were performed in triplicate.

### 2.6. Apparent Solubility of Lupalbigenin in SNEDDS Formulation

An excess quantity of DSE was combined with 500 mg of each anhydrous SNEDDS formulation. After vortex mixing, the samples were maintained at 25 °C for 24 h to allow equilibration. Material that remained unsolubilized was separated by centrifugation at 9170× *g* for 15 min. A 10 µL portion of each supernatant was transferred into 990 µL of methanol, producing a 100-fold analytical dilution, and subsequently passed through a 0.22 µm nylon membrane filter. Lupalbigenin concentrations were quantified using the HPLC procedure described in Section 2.3 and corrected for the dilution factor. The results were reported as the apparent solubility of lupalbigenin per gram of SNEDDS formulation because DSE, rather than purified lupalbigenin, was used in the experiment. The measured responses were entered into the D-optimal models described in Section 2.4.2, and prediction contour plots were generated using MODDE. The final formulation was selected using the combined physicochemical and self-emulsification criteria specified in Section 2.4.2 rather than apparent lupalbigenin solubility alone.

### 2.7. Preparation and Characterization of DSE-SNEDDS and Its NE

DSE was incorporated into the selected SNEDDS to achieve high DSE loading with minimal excipient concentrations, as described above. The droplet size and size distribution of the resulting DSE-loaded NE (DSE-NE) were measured using PCS and compared with those of the blank formulations.

### 2.8. In Vitro Anti-Inflammatory Activity

#### 2.8.1. Cell Culture

Culture conditions for RAW 264.7 macrophages were adapted from a previously reported procedure [28]. The cells were maintained in Dulbecco’s modified Eagle medium (DMEM) supplemented with 1% penicillin–streptomycin (100 µg/mL penicillin and 25 µg/mL streptomycin) and 10% fetal bovine serum (FBS). Cultures were incubated at 37 °C under 95% relative humidity and 5% CO_2_. The experimental protocol was reviewed and approved by the Institutional Biosafety Committee of Naresuan University (approval no. 67-41).

#### 2.8.2. Cell Viability Assay

The effects of DSE dissolved in DMSO (DSE-DMSO), DSE-NE, and blank NE on RAW 264.7 cell viability were examined using an MTT assay adapted from a published procedure [29]. Cells were plated in 96-well plates at 1 × 10^4^ cells/well in 100 µL of complete DMEM and allowed to attach for 24 h at 37 °C under 95% relative humidity and 5% CO_2_. DSE-DMSO was diluted with complete DMEM to obtain final DSE concentrations of 1–100 µg/mL. A vehicle control containing the corresponding concentration of DMSO was included. The DSE-NE stock was prepared by dispersing 500 mg of DSE-NE in FBS-free DMEM to a final volume of 1 mL, giving a formulation concentration of 500,000 µg/mL. Because DSE-NE contained 2% *w*/*w* DSE, this stock was equivalent to 10,000 µg/mL DSE. Working solutions of DSE-NE were prepared according to their DSE-equivalent concentrations to permit comparison with DSE-DMSO. Blank NE was evaluated at excipient concentrations corresponding to those in the DSE-NE treatments.

Following 24 h of exposure, 10 µL of MTT solution prepared in phosphate-buffered saline (5 mg/mL) was added to each well. The plates were incubated for another 3 h, after which the medium was removed and the intracellular formazan was solubilized with 100 µL of DMSO. Absorbance was recorded at 570 nm using a SpectraMax M3 microplate reader (Molecular Devices, San Jose, CA, USA). Cell viability was calculated relative to the appropriate vehicle control as follows:

(1) % Cell viability = (Atest/Avehicle) × 100,

where Atest and Avehicle are the mean absorbance values obtained from the treated and vehicle-control wells, respectively. Concentrations that maintained at least 80% cell viability were considered noncytotoxic and were used in the subsequent NO-production assay. Each condition was analyzed in triplicate wells, and the assay was conducted in at least three independent experiments.

#### 2.8.3. Nitric Oxide (NO) Production Assay

The effect of the test preparations on lipopolysaccharide-induced NO production was evaluated in RAW 264.7 macrophages using a procedure adapted from a previous report [30]. Cells were seeded in 96-well plates at a density of 5 × 10^5^ cells/mL and incubated for 24 h at 37 °C under 95% relative humidity and 5% CO_2_. After removal of the culture medium, the cells were stimulated with lipopolysaccharide (LPS; 2 µg/mL) for 30 min and subsequently exposed to the test preparations for 24 h. DSE-DMSO and DSE-NE were compared at equivalent DSE concentrations selected from the cell-viability results. Blank NE was included at an excipient concentration matching that of the corresponding DSE-NE treatment to account for any contribution from the formulation components. DXA served as the positive anti-inflammatory control. LPS-stimulated cells without sample treatment and unstimulated cells were included as the LPS and negative controls, respectively.

Nitrite accumulation in the culture medium, used as an indirect measure of NO production, was determined using the Griess reaction. The percentage inhibition of NO production was calculated from the absorbance values using Equation (2):

(2) NO inhibition (%) = 100 − [(Atest − ANeg)/(ALPS − ANeg)] × 100,

where Atest represents the mean absorbance of LPS-stimulated cells treated with the test sample, ALPS is the mean absorbance of LPS-stimulated cells without sample treatment, and ANeg is the mean absorbance of unstimulated cells. Each treatment was analyzed in triplicate wells, and the experiment was independently repeated at least three times.

### 2.9. Stability Study

The DSE-SNEDDS and DSE-NE formulations were stored at three different storage temperatures, 4 °C, 25 °C, and 40 °C, for a period of 12 weeks. Physical stability including phase separation, color change, or turbidity appearance was assessed by visual observation. The droplet sizes of stored DSE-NE and DSE-NE prepared from stored DSE-SNEDDS were measured using the PCS method and compared with the droplet size of freshly prepared DSE-NE prior to storage. To evaluate chemical stability, the content of the active ingredient lupalbigenin was analyzed using the HPLC method described above; all tested formulations were diluted with methanol (1:200 weight ratio) prior to HPLC analysis. All experiments were performed in triplicate.

### 2.10. Statistical Analysis

Results are expressed as mean ± SEM. The number and type of replicates are specified in the relevant methods or figure captions. Differences among extraction methods were analyzed using one-way ANOVA followed by Tukey’s post hoc test. The same analysis was applied to comparisons among treatment groups in the cell-viability and NO-production assays. For the stability study, the three storage-temperature groups were compared separately at each sampling time using one-way ANOVA followed by Tukey’s post hoc test. Concentration–response curves were fitted by nonlinear regression using GraphPad Prism version 11 (GraphPad Software, Boston, MA, USA) to estimate the IC_20_ and IC_50_ values. The DoE data were fitted by partial least-squares regression using MODDE. Model fit and predictive ability were assessed using R^2^, Q^2^, regression coefficients, and prediction errors. Differences were considered statistically significant at *p* < 0.05.

## 3. Results and Discussion

### 3.1. Extraction and Chemical Analysis of DSE

Ethanol (95%) was selected as the extraction solvent due to its low toxicity, biodegradability, and wide recognition as a green solvent, particularly in pharmaceutical and cosmetic applications. As a renewable bio-based solvent, ethanol offers a safer and more environmentally friendly alternative to conventional organic solvents such as methanol or chloroform. In addition, preliminary screening and supporting literature indicated that ethanol effectively extracts flavonoids and related phytoconstituents from medicinal plant [31,32,33].

DSE appeared as a pale yellow-brown solid. Ultrasound-assisted extraction of *D. scandens* yielded the highest amount (7.64 ± 0.39% *w*/*w*), calculated as the weight of the dry extract obtained from the dried plant material, followed by maceration with shaking at 200 rpm for 24 h (6.13 ± 0.05% *w*/*w*) and maceration for 24 h without shaking (5.35 ± 0.18% *w*/*w*). The extraction yield obtained from ultrasound-assisted extraction was significantly higher than that from maceration without shaking (*p* < 0.05), but not significantly different from maceration with shaking.

**Figure 3:**
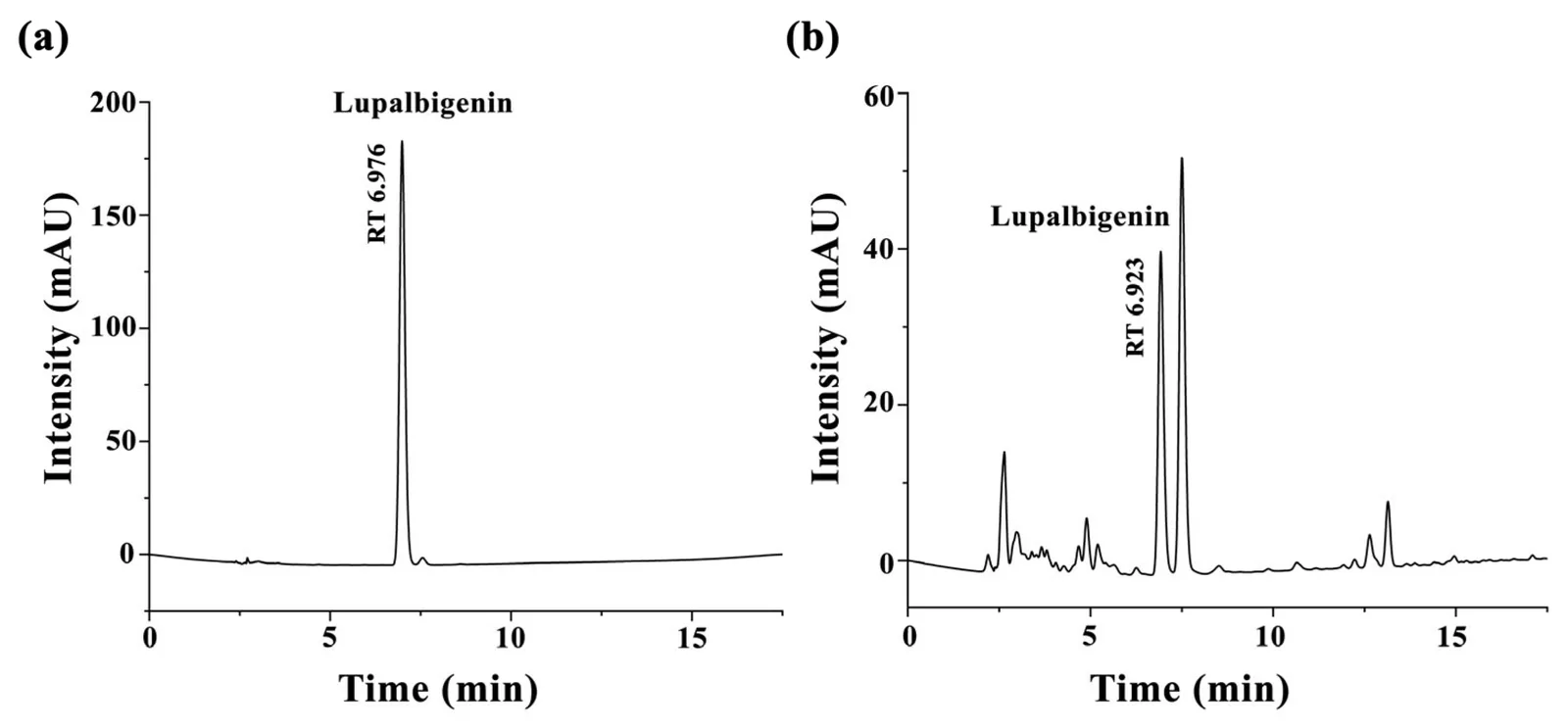
Chromatogram of a standard lupalbigenin (**a**) and DSE (**b**), RT, retention time.

HPLC chromatograms of lupalbigenin and DSE extract are shown in Figure 3. A standard lupalbigenin showed a retention time of 6.976 min (Figure 3a). However, lupalbigenin was detected in DSE chromatogram at 6.923 min (Figure 3b). This minor shift in retention time is considered insignificant and is typically attributed to the matrix effect. The presence of numerous co-extracted compounds in the complex plant extract can subtly alter the interaction between the analyte and the stationary phase, causing this slight variation. The method was validated by estimating a linearity range of 2–100 µg/mL with a coefficient of determination > 0.999 (Figure S1). The LOD and LOQ values for lupalbigenin were 0.5 µg/mL and 2.0 µg/mL, respectively (Table S1). The accuracy of the analytical procedure was established by an average recovery of 104.52% across all three tested concentrations (Table S2 and Table S3). Furthermore, the method demonstrated high precision, yielding relative standard deviations (% RSD) under 0.79% for repeatability and 1.94% for intermediate precision, fulfilling all specified validation thresholds (Tables S4–S6).

Lupalbigenin exhibited a similar extraction yield trend in crude extract. The lupalbigenin content was significantly different (*p* < 0.05) among extraction methods, with values of 108.69 ± 0.12, 84.89 ± 0.09, and 70.79 ± 0.10 µg/mg DSE for ultrasound-assisted extraction, maceration with shaking, and maceration without shaking, respectively. Mechanical shaking resulted in an approximately 20% higher lupalbigenin content than static maceration. This difference may be related to better contact between the plant material and the solvent, which facilitates mass transfer during extraction [34]. Ultrasound-assisted extraction produced the highest crude extract yield and lupalbigenin content, demonstrating greater extraction efficiency than both maceration methods.

### 3.2. Apparent Solubility of Lupalbigenin in Excipients

The ability of an excipient to accommodate the compound of interest is an important consideration when selecting components for a SNEDDS formulation. In this study, lupalbigenin concentrations in the DSE–excipient samples were quantified by HPLC using a calibration curve prepared from standard lupalbigenin. Because the experiments were conducted with crude DSE rather than isolated lupalbigenin, other constituents of the extract may have influenced its solubilization. The results are therefore expressed as the apparent solubility of lupalbigenin from DSE and should not be interpreted as the intrinsic solubility of the pure compound. This measurement was considered relevant to formulation development because DSE was the material incorporated into the SNEDDS.

**Figure 4:**
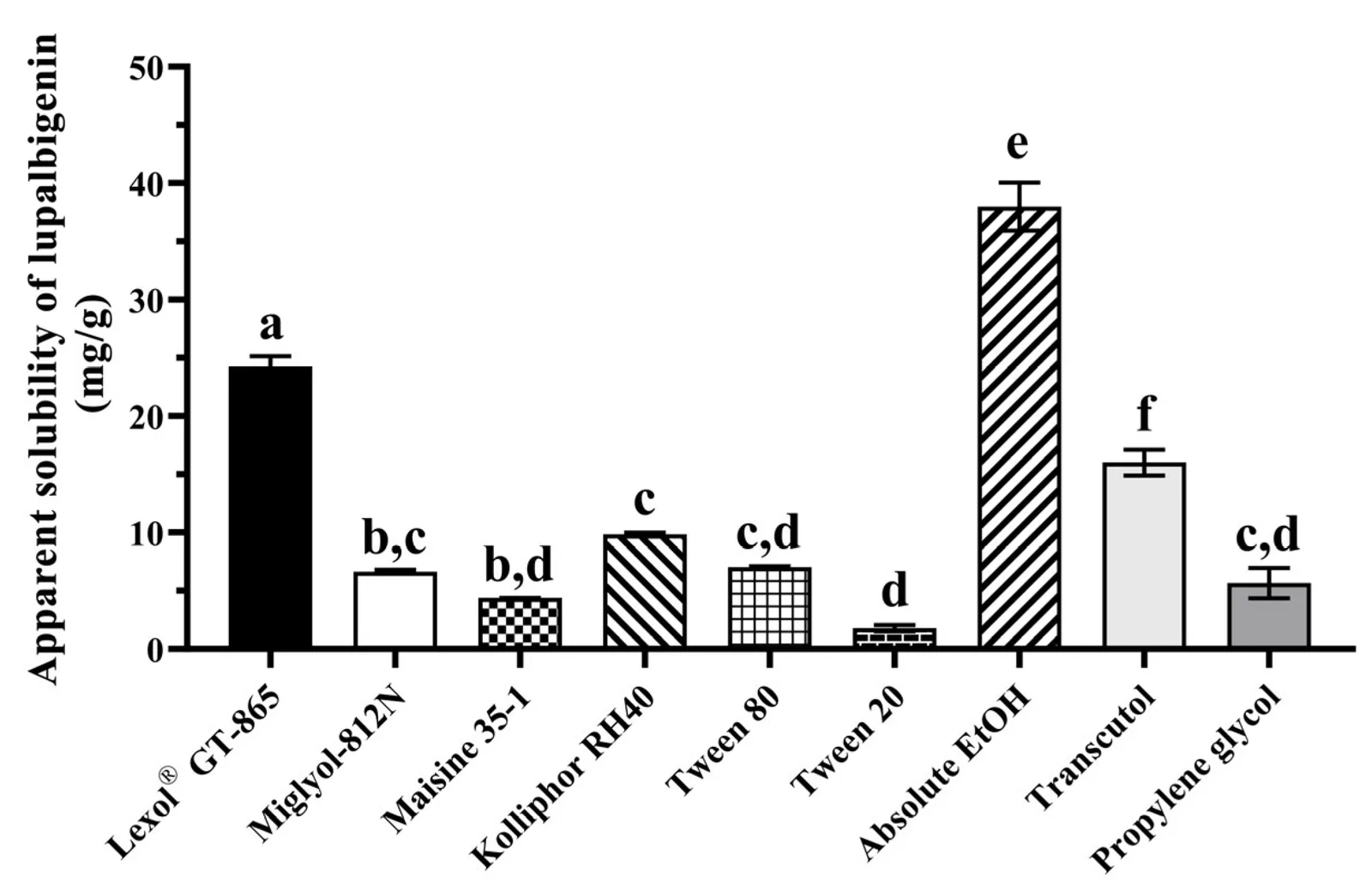
Apparent solubility of lupalbigenin from DSE in the investigated excipients. Data are presented as mean ± SEM from three independent solubility determinations (*n* = 3). Differences among excipients were analyzed using one-way ANOVA followed by Tukey’s post hoc test. Different lowercase letters indicate statistically significant differences (*p* < 0.05).

Lupalbigenin was highly soluble in absolute ethanol (38.00 ± 2.06 mg/g), followed by Lexol^®^ GT-865, an MCT (24.22 ± 0.92 mg/g), and Transcutol, a co-surfactant (16.01 ± 1.10 mg/g). The high apparent solubility of lupalbigenin in absolute ethanol, Lexol^®^ GT-865, and Transcutol can be explained by the Hansen solubility parameter (HSP) which is commonly used to assessed the miscibility between two compounds [35]. Solvents with HSP values similar to those of the solute are classified as good solvents. The HSP values for lupalbigenin, absolute ethanol, Lexol^®^ GT-865, and Transcutol were 26.70, 26.50, 19.21, and 21.40, respectively [35,36]. The highest apparent solubility of lupalbigenin in absolute ethanol as a good solvent is attributed to similar HSP values. HSP describes the intermolecular forces between molecules using three parameters, such as dispersion (δD), hydrogen-bonding interaction (δH), and polar contribution (δP); thus, HSPs are effective tools for assessing how molecules interact and dissolve in various simple solvents [37,38]. Due to the large molecules of medium-chain triglyceride of Lexol^®^ GT-865 MB, the preference of lipophilicity affects the solubility. LogP (Octanol-Water partition coefficient) reflects the lipophilicity of a molecule, was used to predict the behavior of compounds in environmental systems. Despite Transcutol having an HSP closer to lupalbigenin than Lexol^®^ GT-865, lupalbigenin exhibits higher solubility in Lexol^®^ GT-865. This result is influence by the partition coefficient (Log P) of Lexol^®^ GT-865, an MCT (Log P = 6), which is higher than that of Transcutol (Log P = −0.5) [39]. In addition, the surfactant that provided the highest solubilization capacity for lupalbigenin was Kolliphor RH40 (9.82 ± 0.17 mg/g), followed by Tween 80 (7.02 ± 0.09 mg/g), and Tween 20 (1.78 ± 0.27 mg/g). The solubility tests in this study suggest that high solubility of lupalbigenin can be obtained with absolute ethanol, Lexol^®^ GT-865, Transcutol, Kolliphor RH40, and Tween80 as shown in Figure 4.

Previous pharmaceutical and cosmetic use, together with the available safety data, was also considered when selecting the excipients. Ethanol is commonly used in topical antiseptic preparations, although its potential to cause skin irritation depends on its concentration and the duration of exposure [40,41]. Lexol^®^ GT-865 is a caprylic/capric triglyceride that is widely used as an emollient and is considered safe under current conditions of cosmetic use [42]. Nonclinical safety studies of high-purity Transcutol have reported a low potential for dermal irritation [43]. Safety assessments of PEGylated castor oils, including PEG-40 hydrogenated castor oil (Kolliphor RH40), reported that the available clinical data were generally negative for irritation and sensitization [43]. Polysorbate 80 is a nonionic, biocompatible surfactant that has been investigated for the preparation of microemulsion systems for pharmaceutical processing [44]. However, these reports concern the individual excipients and cannot confirm the dermal safety of the final DSE-SNEDDS or DSE-NE formulation. The excipients were therefore selected primarily for their ability to solubilize lupalbigenin and their functions in SNEDDS formation, while the existing safety information was considered supportive for future topical formulation development.

### 3.3. Preparation and Characterization of SNEDDS Formulations

The excipients selected from the apparent solubility study were used to create an experimental matrix for preparing SNEDDS formulations. As shown in Table 1, 24 SNEDDS formulations were generated using a DoE, specifically a D-optimal design, for selecting the most suitable SNEDDS formulation. These formulations were categorized into two groups: one group of Tween 80 systems (FT1–FT12) and another group of Kolliphor RH40 systems (FC1–FC12). Once the components had been combined, all anhydrous formulations appeared clear and uniform, with no visible phase separation. Their behavior following aqueous dilution was subsequently examined, as a suitable SNEDDS should form and maintain a uniform dispersion in an aqueous environment. The occurrence of droplet aggregation, a cream layer, oil separation, or complete emulsion breakdown was considered evidence of physical instability [45]. In this study, dispersing the formulated SNEDDS in Milli-Q water at a 1:100 ratio at ambient temperature resulted in mixtures that could be categorized into five grades: A, B, C, D, and E [27]. Grade A formulations were the most effective in forming the desired NE, characterized by a clear or bluish appearance and rapid formation within 1 min. This includes the MCT with Kolliphor RH40 formulation, FC2.

Particle characterization was performed after diluting each formulated SNEDDS in water at a 1:100 ratio, which was determined to be optimal. At this stage, the purpose was to screen and optimize the NE without the addition of the extract, in order to select the most stable base formulation prior to incorporating the active ingredient. As expected, the results indicated that the droplet size of the formulations correlated with their grading. In addition, for the formulations classified as Grade D or Grade E emulsions according to the self-emulsification performance grading system (mention above), droplet size could not be determined due to phase separation and is expressed as “ND” in the table. The formulations in grade A, particularly FC2, demonstrated a small droplet size of approximately 100 nm. droplet size distribution is expressed in terms of the polydispersity index (PdI). Experimental results revealed that these formulations exhibited low PdI values (not exceeding 0.20), indicating a relatively high degree of droplet size uniformity within the system [46]. Based on these results, it can be concluded that the best spontaneously formed transparent NE was obtained from the SNEDDS formulation coded FC2, which exhibited uniform droplet distribution with a size of 100 nm.

### 3.4. Apparent Solubility of Lupalbigenin in SNEDDS Formulations

**Figure 5:**
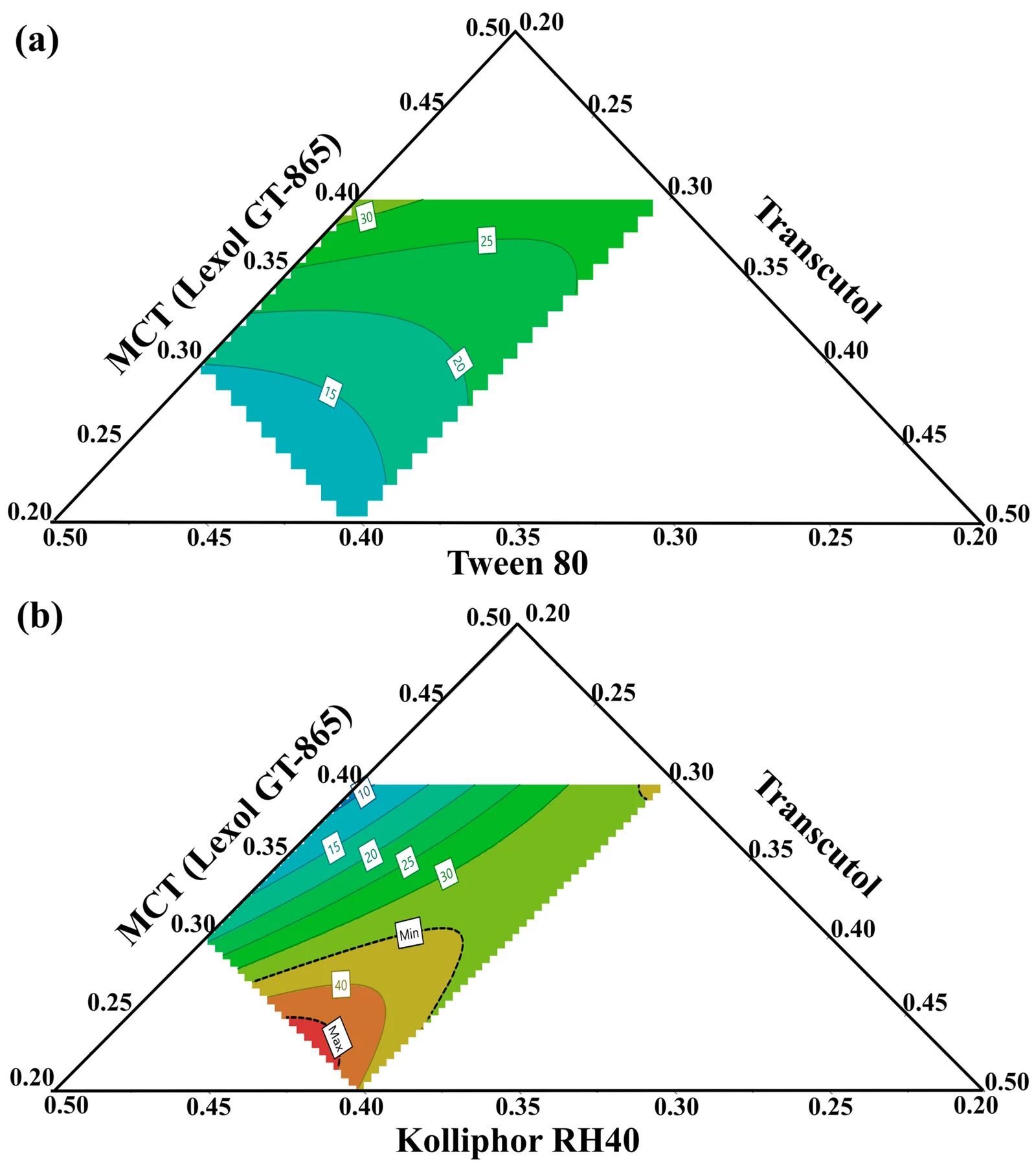
Prediction contour plots of apparent lupalbigenin solubility in DSE-SNEDDS formulations: Tween80 SNEDDS system (**a**) and Kolliphor RH40 SNEDDS system (**b**). Solubility levels are indicated as: ■ ≥45 mg/g, ■ 40–45 mg/g, ■ 35–40 mg/g, ■ 30–35 mg/g, ■ 25–30 mg/g, ■ 20–25 mg/g, ■ 15–20 mg/g, ■ 10–15 mg/g.

A D-optimal design was used to examine how the proportions of the formulation components affected the apparent solubility of lupalbigenin and to identify a suitable composition for DSE-SNEDDS. This approach enables formulation variables to be evaluated systematically while reducing the number of experimental runs required [47]. Because DSE, rather than purified lupalbigenin, was incorporated into the formulations, the measured response represents the apparent solubility of lupalbigenin in the presence of other extract constituents and should not be interpreted as the intrinsic solubility of the pure compound. The experimental responses and corresponding formulation compositions were used to construct prediction contour plots for the Tween 80 and Kolliphor RH40 systems (Figure 5). Across the 30 formulations listed in Table 2, the mean absolute prediction error was 5.52%. Twenty-three formulations had absolute prediction errors below 10%, whereas the remaining seven had errors ranging from 10.14% to 26.71%. All six additional validation formulations (ET1–ET3 and EC1–EC3) had absolute prediction errors below 10%, ranging from 0.44% to 5.04%. The validation results support the predictive performance of the models within the investigated design space; however, the larger errors obtained for some design formulations indicate that prediction accuracy varied across the tested compositions.

**Table 2:** Lupalbigenin solubility in the tested SNEDDS formulations.

Formulations	Apparent Solubility of Lupalbigenin in SNEDDS (mg/g)		
	Observed	Predicted (Lower-Upper)	Prediction Error (%)
FT1	20.74 ± 0.64	26.28 (20.15–34.26)	26.71
FT2	12.45 ± 0.03	12.80 (9.81–16.68)	2.81
FT3	29.93 ± 0.04	33.75 (25.59–44.49)	12.76
FT4	16.22 ± 0.04	14.89 (11.30–19.64)	8.20
FT5	30.57 ± 0.08	27.32 (22.00–33.93)	10.63
FT6	10.26 ± 0.14	11.30 (9.09–14.03)	10.14
FT7	27.63 ± 0.11	25.37 (20.54–31.35)	8.18
FT8	21.11 ± 0.06	17.71 (14.33–21.87)	16.11
FT9	18.84 ± 0.09	19.58 (17.09–22.45)	3.93
FT10	18.84 ± 0.09	19.58 (17.09–22.45)	3.93
FT11	20.08 ± 0.36	19.58 (17.09–22.45)	2.49
FT12	20.08 ± 0.36	19.58 (17.09–22.45)	2.49
FC1	35.29 ± 0.06	36.11 (31.05–42.00)	2.32
FC2	40.59 ± 0.06	39.23 (33.73–45.62)	3.35
FC3	8.69 ± 0.08	8.45 (7.22–9.89)	2.76
FC4	18.58 ± 0.05	18.54 (15.84–21.69)	0.22
FC5	21.80 ± 0.05	24.11 (21.31–27.27)	10.60
FC6	45.42 ± 0.05	44.83 (39.63–50.71)	1.30
FC7	36.92 ± 0.08	32.68 (28.98–36.86)	11.48
FC8	32.61 ± 0.11	34.06 (30.20–38.42)	4.45
FC9	29.79 ± 0.07	30.04 (27.79–32.46)	0.84
FC10	30.11 ± 0.06	30.04 (27.79–32.46)	0.23
FC11	29.77 ± 0.01	30.04 (27.79–32.46)	0.91
FC12	29.19 ± 0.03	30.04 (27.79–32.46)	2.91
ET1	11.26 ± 1.63	11.31 (9.13–14.02)	0.44
ET2	23.43 ± 1.71	22.25 (17.43–28.39)	5.04
ET3	16.79 ± 2.40	16.35 (14.22–18.81)	2.62
EC1	48.44 ± 2.71	48.01 (42.49–54.25)	0.89
EC2	33.42 ± 1.21	32.05 (27.90–36.82)	4.10
EC3	39.57 ± 2.28	38.44 (35.50–41.62)	2.86

**Figure 6:**
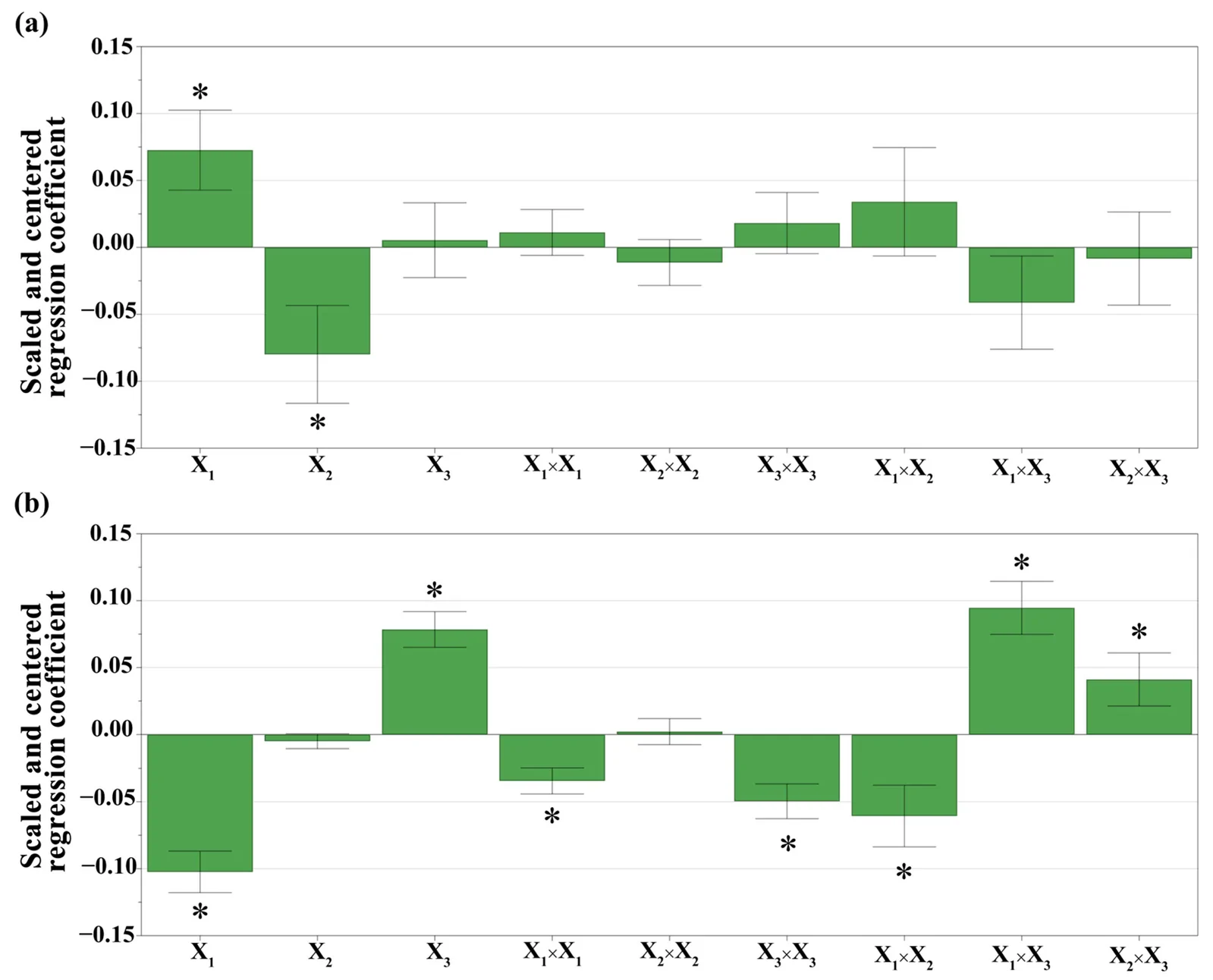
Plots of scaled and centered regression coefficients for the quadratic models describing the apparent solubility of lupalbigenin in DSE-SNEDDS formulations: the Tween 80 system (**a**) and the Kolliphor RH40 system (**b**). Positive and negative coefficients indicate the direction of each model term’s effect on the response. Asterisks indicate statistically significant coefficients (*p* < 0.05).

The coefficients in the model quantified the magnitude and direction (positive or negative) of each term’s influence on the response variable, as well as their statistical significance, as illustrated in Figure 6. Lexol^®^ GT-865 (*X*_1_) and Tween 80 (*X*_2_) were identified as the most influential variables affecting lupalbigenin solubility in the SNEDDS formulations within the Tween 80 system group (FT1–FT12), based on coefficient significance. In contrast, for the SNEDDS formulations within the Kolliphor RH40 system group (FC1–FC12), the key variables included Lexol^®^ GT-865 (*X*_1_), Transcutol (*X*_3_), *X*_1_^2^, *X*_3_^2^, *X*_1_*X*_2_, *X*_1_*X*_3_, and *X*_2_*X*_3_.

Furthermore, Q^2^ and R^2^ values closest to 1 with logarithmic transformation of the data set of FT1–FT12 and FC1–FC12, indicated a good fit (R^2^ = 0.925 and 0.986, respectively) and strong prediction power (Q^2^ = 0.748 and 0.764, respectively). The second-order polynomial equations for the lupalbigenin solubility values in the SNEDDS formulations for the Tween 80 systems (Equation (3)) and Kolliphor RH40 systems (Equation (4)) were derived as follows:

(3) Y (Lupalbigenin solubility, mg/g) = 8.92749 + 0.50201*X*_1_ − 0.55263*X*_2_ + 0.03701*X*_3_ − 0.07734*X*_1_^2^ −0.07817*X*_2_^2^ + 0.12540*X*_3_^2^ + 0.23543*X*_1_*X*_2_ − 0.28521*X*_1_*X*_3_ − 0.05782*X*_2_*X*_3_

(4) Y (Lupalbigenin solubility, mg/g) = 7.87244 − 0.53449*X*_1_ − 0.02587*X*_2_ + 0.40968*X*_3_ −0.17959*X*_1_^2^ + 0.01131*X*_2_^2^ − 0.25914*X*_3_^2^ − 0.31653*X*_1_*X*_2_ + 0.49402*X*_1_*X*_3_ + 0.21488*X*_2_*X*_3_

where *X*_1_ = lipid (Lexol^®^ GT-865); *X*_2_ = surfactant (Tween 80 or Kolliphor RH40); *X*_3_ = co-surfactant (Transcutol). In addition, three further experiments for each surfactant system (ET1–ET3 and EC1–EC3) were conducted to validate this model. The results showed that errors in prediction and observation of all six external validation formulations were less than 10%, suggesting that this model may be used effectively to optimize and determine the relevant variables.

### 3.5. Preparation and Characterization of DSE Loaded SNEDDS and Its NE

The SNEDDS formulation FC2 was selected for further study due to its superior ability to solubilize lupalbigenin and its classification as grade A. The optimization strategy was designed to balance both drug loading capacity and emulsification efficiency. While solubility is a critical parameter, the ability of a formulation to spontaneously form a fine and stable NE plays a more critical role in ensuring consistent physicochemical behavior. Although FC6 exhibited slightly higher lupalbigenin solubility, FC2 was ultimately selected as the optimal formulation due to its superior self-emulsification performance (Grade A) and sufficient drug-loading capacity, which together are expected to enhance overall formulation stability and effectiveness. Following aqueous dilution, the formulation self-emulsified within 1 min and produced a clear dispersion with a slight bluish appearance. Among the formulations examined, the resulting NE had the lowest mean droplet size and PdI, indicating a relatively uniform droplet-size distribution. Consequently, DSE was incorporated into the SNEDDS formulation FC2 to achieve a final concentration of 10% *w*/*w* DSE. Moreover, the resulting DSE-NE, prepared with 10% *w*/*w* DSE in the formulation, was subsequently diluted five-fold with water. The selection of the 1:5 dilution was based on preliminary optimization studies of the DSE-SNEDDS preconcentrate, in which various dilution ratios (1:1, 1:2, 1:3, 1:4, and 1:5) were evaluated. The optimal condition was determined based on physical stability (i.e., absence of phase separation) and droplet characteristics, including droplet size within the nanometer range (≤200 nm) and narrow size distribution. Among the tested conditions, the 1:5 dilution yielded the most stable nanoemulsion with desirable physicochemical properties and was therefore selected for use in biological assays. The preformed DSE-NE obtained under this condition, rather than the DSE-SNEDDS preconcentrate, was the formulation intended for subsequent topical evaluation. Thus, the preconcentrate was not intended for direct application to the skin or to self-emulsify using the limited amount of water present on the skin surface. Prior to PCS measurement, an aliquot of the resulting DSE-NE was further diluted 100-fold with water. The measured mean droplet size and PdI were 172.5 ± 0.3 nm and 0.31 ± 0.01, respectively.

### 3.6. Cytotoxicity and In Vitro Anti-Inflammatory Activity

**Figure 7:**
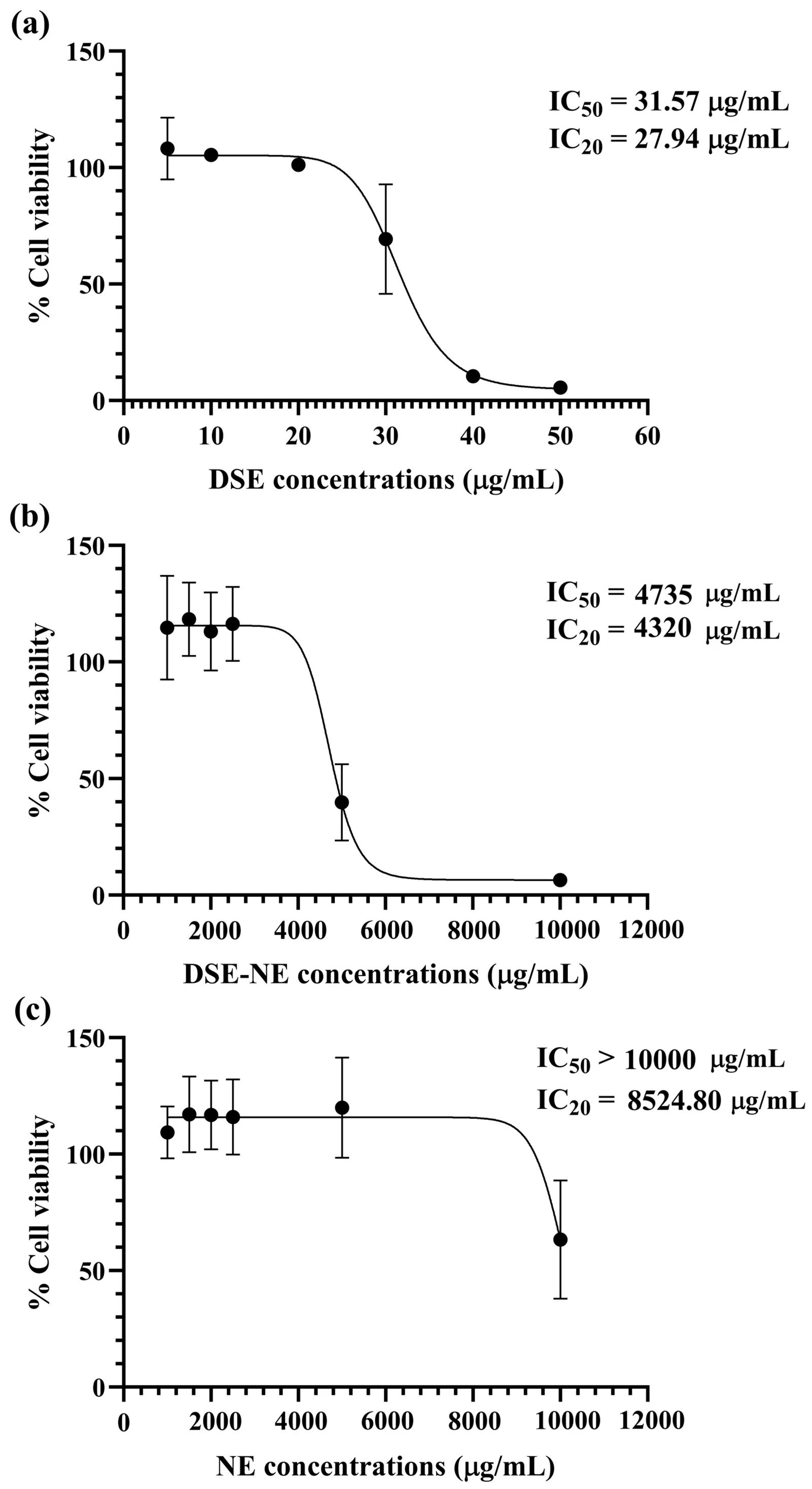
Dose–response curves of RAW 264.7 macrophage cell viability exposed to DSE-DMSO (**a**), DSE-NE (**b**), and NE (**c**). All assays were conducted in serum-free DMEM (without FBS) containing 1% penicillin-streptomycin. Free DSE was evaluated with a final DMSO concentration of <1% *v*/*v* as the vehicle control. Data are expressed as mean ± SEM (n = 3). DSE-NE concentrations reflect total formulation weight (2% *w*/*w* DSE loading).

The cytotoxicity test results shown in Figure 7 indicate that the viability of RAW 264.7 macrophages decreased slightly as the DSE concentration increased. At the same concentration levels, DSE-NE exhibited lower cytotoxicity compared to the DSE solution in DMSO (DSE-DMSO).

The IC_50_ values for RAW 264.7 macrophage cells exposed to DSE-DMSO and DSE-NE were 31.57 µg DSE/mL and 4735 µg formulation/mL, respectively, with the latter corresponding to 94.70 µg DSE/mL based on the DSE content of the formulation (Figure 7a,b). An IC_50_ value could not be determined for the blank NE because cell viability did not decrease to 50% within the tested concentration range. Similarly, the IC_20_ values for RAW 264.7 cells exposed to DSE-DMSO and DSE-NE were 27.94 µg DSE /mL and 4320 µg formulation/mL, respectively, with 4320 µg formulation/mL corresponding to 86.40 µg DSE/mL of DSE in the DSE-NE formulation. The IC_20_ value for the blank NE (NE without DSE) was 8524.80 µg formulation/mL (Figure 7c). The reduced cytotoxicity observed for DSE-NE may be partly related to the ability of NE formulations to encapsulate plant extracts, thereby modulating the release of active compounds and preventing sudden exposure to high concentrations. This controlled release may help mitigate direct cytotoxic effects. For instance, a study on turmeric extract-loaded NE demonstrated that the formulation did not exhibit significant cytotoxicity across various cell lines, suggesting that NE formulations provide a safer delivery system for bioactive compounds [48]. However, release behavior was not directly investigated in the present study, and the mechanism underlying the lower cytotoxicity of DSE-NE remains to be confirmed.

**Figure 8:**
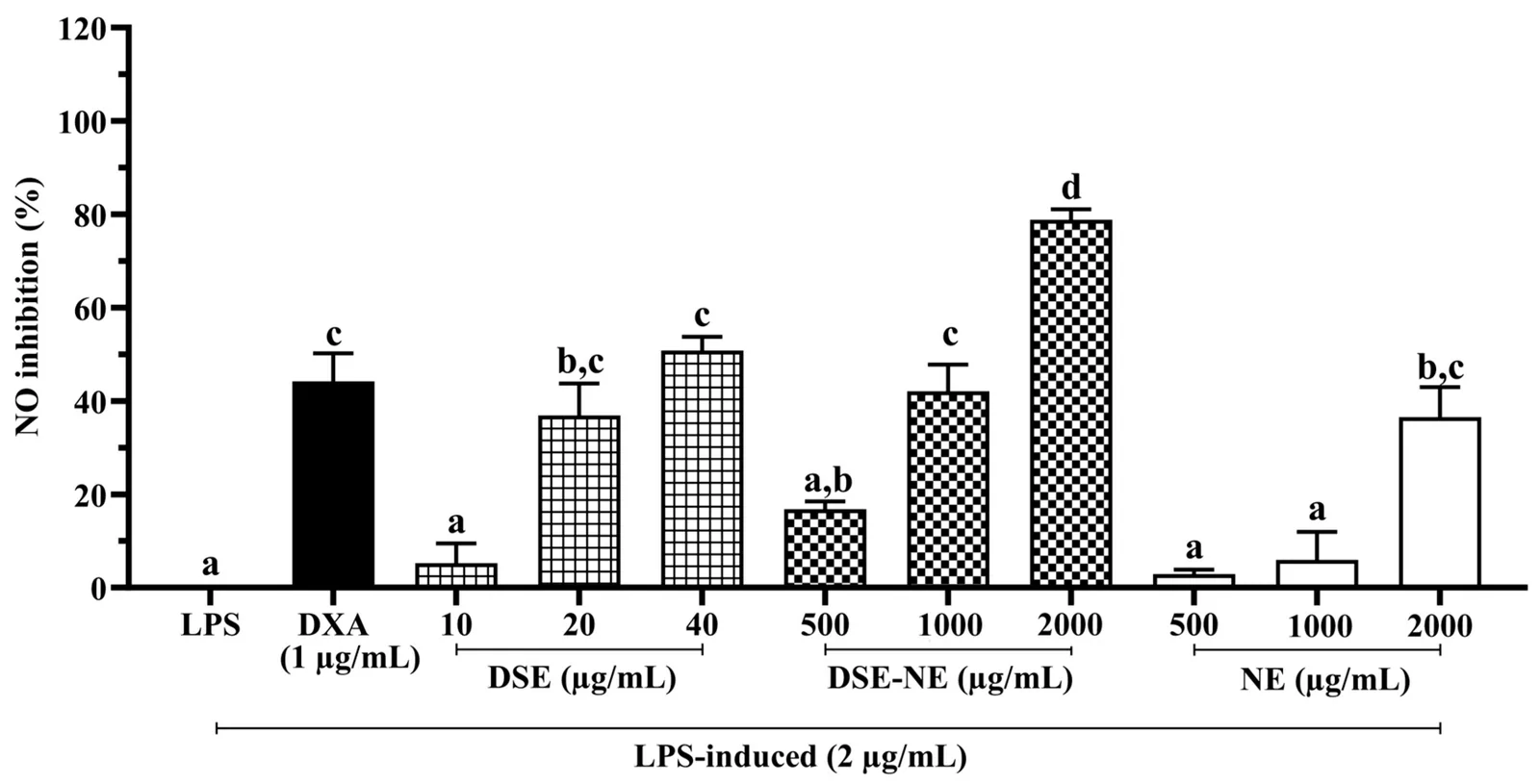
Effects of DXA (1 µg/mL), DSE, DSE-NE, and NE on LPS-induced (2 µg/mL) NO production in RAW 264.7 cells. Treatments were prepared in serum-free DMEM supplemented with 1% penicillin-streptomycin. DSE-NE concentrations of 500, 1000, and 2000 µg/mL correspond to actual DSE concentrations of 10, 20, and 40 µg/mL, respectively. Different letters indicate significant differences between groups at *p* < 0.05.

NO serves as a key mediator in the inflammatory response of RAW 264.7 macrophage cells. Upon stimulation with pro-inflammatory agents such as LPS, these cells markedly upregulate NO production, thereby contributing to the inflammatory process. Consequently, inhibition of NO production in LPS-stimulated RAW 264.7 cells is widely employed as a standard in vitro assay for evaluating the anti-inflammatory potential of bioactive compounds [49]. In the present study, both DSE-DMSO and DSE-NE effectively reduced NO accumulation in LPS-stimulated RAW 264.7 cells at equivalent DSE concentrations. However, at a higher concentration of 2000 µg formulation/mL DSE-NE, corresponding to 40 µg/mL of DSE based on formulation content, DSE-NE exhibited a significantly greater inhibitory effect on NO accumulation as shown in Figure 8 Specifically, DSE-NE inhibited NO production by 78.79 ± 2.31%, while DSE and Blank NE resulted in 50.78 ± 2.95% and 36.53 ± 6.45% inhibition, respectively. Thus, DSE-NE produced greater inhibition than either DSE-DMSO or blank NE when each was tested separately.

However, the present experimental design does not establish whether the contributions of DSE and the nanoemulsion vehicle were additive or synergistic. This enhanced effect may be attributed to the NE system, which improves the solubility and stability of plant-derived compounds, thereby facilitating more efficient cellular uptake and interaction. Such enhancements can lead to improved therapeutic efficacy and reduced toxicity. For instance, a previous study demonstrated that date palm extract loaded NE exhibited greater cytotoxic activity against cancer cell lines compared to its the free extract counterpart, suggesting improved bioavailability and biological activity of the nanoformulated extract [50].

Interestingly, the NE without DSE also inhibited NO production by 36.53 ± 6.45%, likely due to the intrinsic effects of the excipients, particularly Kolliphor RH40, which has been reported to exert mild anti-inflammatory effects in macrophage models [51,52]. This finding highlights the importance of including blank NE control and further supports that the pronounced NO inhibition effect observed with DSE-NE is attributable to the combined contributions of the active phytoconstituents and the NE carrier system, rather than the extract alone. The unstimulated control was used to correct for basal nitrite levels in the calculation of NO inhibition and was therefore not presented as an inhibitory treatment. The Griess assay measured nitrite accumulation as an indirect indicator of NO production; cytokine concentrations were not determined in this study. Accordingly, the findings were interpreted specifically as inhibition of LPS-induced NO production.

### 3.7. Stability Study of DSE-NE

**Figure 9:**
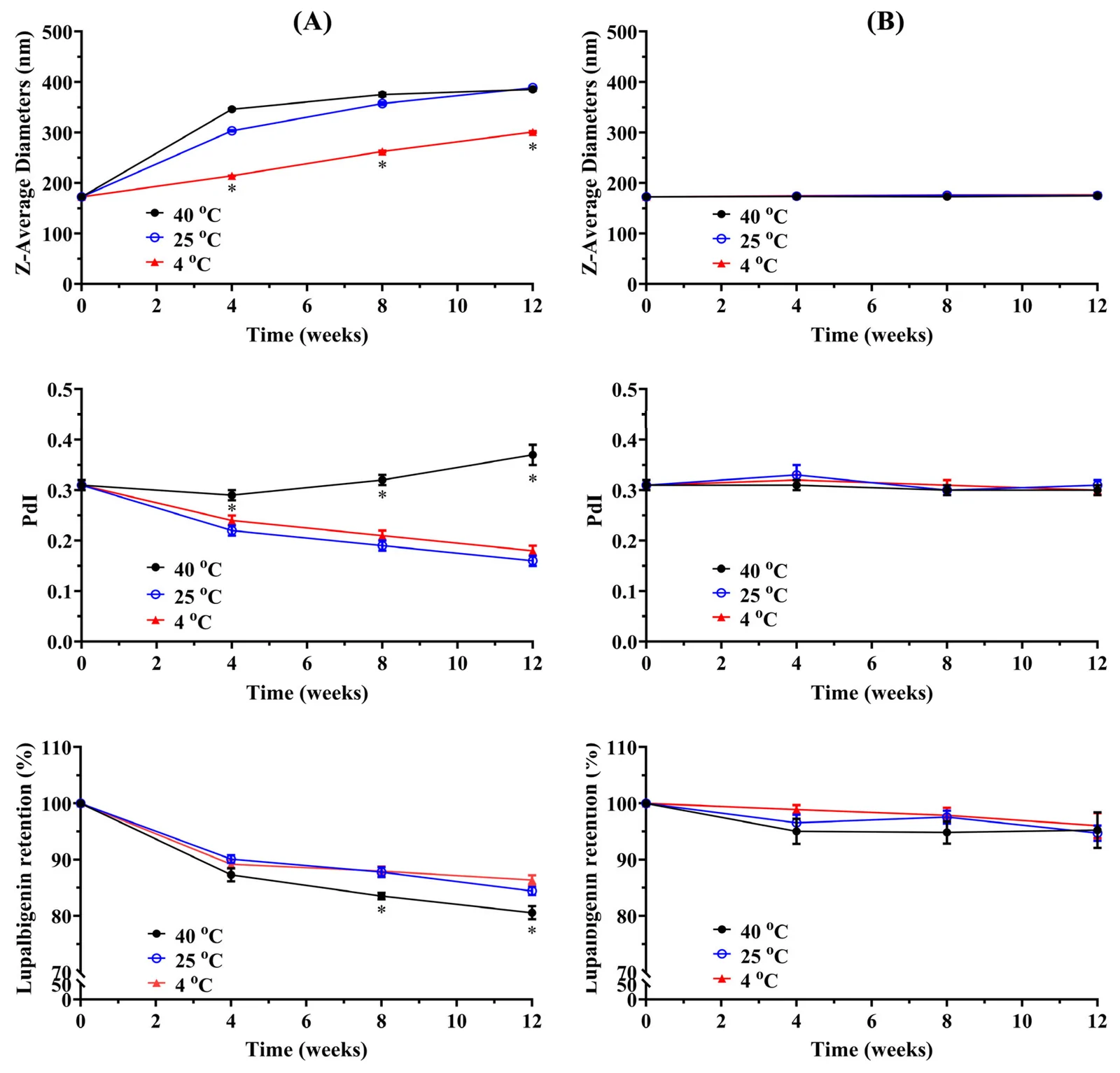
Mean droplet diameter (Z-average), PdI, and lupalbigenin retention measured during 12 weeks of storage at 4, 25, and 40 °C: preformed DSE-NE stored in its aqueous form (**A**) and DSE-NE freshly prepared at each sampling time from the stored DSE-SNEDDS preconcentrate (**B**). Data are presented as mean ± SEM (*n* = 3). An asterisk indicates a significant difference among the storage-temperature groups at the corresponding time point (*p* < 0.05).

The stability of both the DSE-SNEDDS preconcentrate and its corresponding DSE-NE was evaluated over 12 weeks under different storage conditions, and the results are presented in Figure 9. Throughout the study period, neither the DSE-SNEDDS preconcentrate nor the preformed DSE-NE exhibited phase separation or visible turbidity. Since droplet size is an important indicator of NE stability, changes in droplet size were monitored during storage at 4 °C, 25 °C, and 40 °C. To distinguish the influence of storage in different dosage forms, two storage conditions were investigated: (i) DSE-SNEDDS preconcentrate stored for 4, 8, and 12 weeks and subsequently diluted with water immediately before analysis to produce fresh DSE-NE, and (ii) preformed DSE-NE stored in its diluted state for the same period. No noticeable color change was observed in either formulation except for a slight dark-yellow discoloration in the DSE-SNEDDS preconcentrate stored at 40 °C for 12 weeks. Freshly prepared DSE-NE generated from the stored DSE-SNEDDS preconcentrate maintained a nearly constant droplet size throughout the study, with no significant differences observed under any storage condition. In contrast, the preformed DSE-NE exhibited a significant increase in droplet size after prolonged storage. After 12 weeks at 40 °C, the average droplet size increased from 172.5 nm to approximately 390 nm Although no phase separation or visible turbidity was observed, the increase in mean droplet size indicated that the preformed DSE-NE underwent physical changes during storage. The PdI remained below 0.4 throughout the study. Changes in droplet size were smaller at 4 °C than at 25 or 40 °C. The temperature-dependent droplet growth may be associated with increased molecular mobility and processes such as Ostwald ripening or droplet coalescence [53], although these mechanisms were not investigated directly.

The chemical stability of the formulations was evaluated using lupalbigenin, the principal bioactive marker of DSE. After 12 weeks of storage, the preformed DSE-NE retained approximately 87%, 85%, and 81% of its initial lupalbigenin content at 4 °C, 25 °C, and 40 °C, respectively. Lupalbigenin retention decreased as the storage temperature increased. By comparison, DSE-NE freshly prepared from the stored DSE-SNEDDS preconcentrate retained approximately 96%, 95%, and 95% of its initial lupalbigenin content at 4, 25, and 40 °C, respectively. Lupalbigenin retention was consistently higher when the formulation was stored as the DSE-SNEDDS preconcentrate than when it was stored as the preformed DSE-NE. This difference may be related to the absence of an aqueous phase during preconcentrate storage. However, degradation products were not investigated; therefore, the mechanism responsible for the observed loss of lupalbigenin could not be confirmed.

Under the conditions examined, storage as the DSE-SNEDDS preconcentrate resulted in smaller changes in droplet size and higher lupalbigenin retention than storage as the preformed DSE-NE. The stored preconcentrate also retained its ability to form DSE-NE after aqueous dilution. These results support the selection of the preconcentrate as the more suitable storage form for subsequent formulation development. However, this 12-week study was intended only to compare the two storage forms and was not designed to establish product shelf life or confirm the stability of a finished pharmaceutical formulation. Longer-term studies using a validated stability-indicating method will be required for the final topical dosage form.

## 4. Conclusions

Ultrasound-assisted extraction produced DSE with the highest extract yield and lupalbigenin content, and the resulting extract was used for subsequent SNEDDS development. DoE-based formulation screening identified a DSE-SNEDDS preconcentrate that formed DSE-NE with a mean droplet size of 172.5 ± 0.3 nm and a PdI of 0.31 ± 0.01 after controlled aqueous dilution. At an equivalent DSE concentration of 40 µg/mL, DSE-NE inhibited NO production by 78.79 ± 2.31%, whereas DSE-DMSO produced 50.78 ± 2.95% inhibition. However, the blank NE also inhibited NO production by 36.53 ± 6.45%. The activity of DSE-NE therefore cannot be attributed to DSE alone and may result from contributions from both the extract and the formulation excipients. During the 12-week stability study, storage as the anhydrous DSE-SNEDDS preconcentrate preserved lupalbigenin content and self-emulsifying performance more effectively than storage as the preformed DSE-NE. The comparison of these two storage forms is a practical contribution of this study, as it shows that DSE-SNEDDS is better stored before aqueous dilution. Based on the current findings, DSE-SNEDDS should be considered a precursor for further topical formulation development rather than a finished topical product. Further studies should include comprehensive phytochemical profiling, quantification of multiple marker compounds, and assessment of additional inflammatory biomarkers, together with characterization of the final topical dosage form. Comparisons with free DSE and an appropriate conventional topical formulation, as well as release testing, ex vivo skin permeation and deposition, dermal safety, and in vivo efficacy studies, will also be required.

## Figures and Tables

**Table 1:** Composition of SNEDDS formulations designed using DoE via D-Optimal MODDE software and the effects of different concentrations on self-emulsification efficiency, droplet size, and size distribution (PdI) of the resulting NE.

Formula	Excipient (% *w*/*w*)					Emulsification Time (min)	Droplet Size (nm)	PdI
	(*X*_1_) Lexol GT-865	(*X*_2_)		(*X*_3_) Transcutol	(*X*_4_) Absolute Ethanol			
		Tween 80	Kolliphor RH40					
FT1	40.00	20.00	-	30.00	10.00	E	ND	ND
FT2	20.00	40.00	-	30.00	10.00	B	145.5 ± 0.5	0.18 ± 0.01
FT3	40.00	30.00	-	20.00	10.00	E	ND	ND
FT4	30.00	40.00	-	20.00	10.00	D	ND	ND
FT5	40.00	25.00	-	25.00	10.00	E	ND	ND
FT6	25.00	40.00	-	25.00	10.00	D	ND	ND
FT7	35.00	25.00	-	30.00	10.00	E	ND	ND
FT8	25.00	35.00	-	30.00	10.00	D	ND	ND
FT9	32.50	32.50	-	25.00	10.00	D	ND	ND
FT10	32.50	32.50	-	25.00	10.00	D	ND	ND
FT11	32.50	32.50	-	25.00	10.00	D	ND	ND
FT12	32.50	32.50	-	25.00	10.00	D	ND	ND
FC1	40.00	-	20.00	30.00	10.00	D	ND	ND
FC2	20.00	-	40.00	30.00	10.00	A	103.9 ± 0.3	0.17 ± 0.01
FC3	40.00	-	30.00	20.00	10.00	D	ND	ND
FC4	30.00	-	40.00	20.00	10.00	C	190.8 ± 0.4	0.21 ± 0.01
FC5	40.00	-	25.00	25.00	10.00	D	ND	ND
FC6	25.00	-	40.00	25.00	10.00	B	147.8 ± 0.1	0.17 ± 0.01
FC7	35.00	-	25.00	30.00	10.00	D	ND	ND
FC8	25.00	-	35.00	30.00	10.00	C	172.5 ± 0.8	0.19 ± 0.01
FC9	32.50	-	32.50	25.00	10.00	C	196.0 ± 0.2	0.25 ± 0.01
FC10	32.50	-	32.50	25.00	10.00	C	204.4 ± 2.6	0.26 ± 0.01
FC11	32.50	-	32.50	25.00	10.00	C	197.2 ± 0.8	0.25 ± 0.01
FC12	32.50	-	32.50	25.00	10.00	C	191.9 ± 0.5	0.24 ± 0.01
ET1	23.00	40.00	-	27.00	10.00	C	177.2 ± 1.6	0.22 ± 0.01
ET2	30.00	30.00	-	30.00	10.00	D	ND	ND
ET3	27.50	35.00	-	27.50	10.00	D	ND	ND
EC1	23.00	-	40.00	27.00	10.00	B	113.1 ± 0.4	0.19 ± 0.01
EC2	30.00	-	30.00	30.00	10.00	C	182.2 ± 1.0	0.29 ± 0.01
EC3	27.50	-	35.00	27.50	10.00	C	166.8 ± 1.0	0.18 ± 0.01

Note: ND = not determined and *X*_1_ = lipid; *X*_2_ = surfactant; *X*_3_ = co-surfactant, *X*_4_ = co-solvent.

## Data Availability

Data presented in this study is contained within the article and Supplementary Materials. Further inquiries can be directed to the corresponding author.

## Supplementary Materials

The following supporting information can be downloaded at https://www.mdpi.com/article/10.3390/pharmaceutics18091188/s1, Figure S1: A standard calibration curve demonstrating the correlation between lupalbigenin concentration and its peak area; Table S1: Signal-to-noise data at the experimentally assigned LOD and LOQ; Table S2: Individual measurements used in the lupalbigenin recovery assessment; Table S3: Summary statistics for the recovery results; Table S4: Repeatability of the HPLC determination of lupalbigenin (*n* = 3 per level); Table S5: Individual measurements collected for the intermediate-precision study; Table S6: Intermediate-precision statistics calculated across three analytical days.

## Abbreviations

The following abbreviations are used in this manuscript:

ANOVA	Analysis of Variance
DMEM	Dulbecco’s Modified Eagle Medium
DMSO	Dimethyl sulfoxide
DoE	Design of Experiment
DSE	*Derris scandens* extract
DSE-DMSO	*Derris scandens* extract dissolved in dimethyl sulfoxide
DSE-NE	*Derris scandens* extract-loaded nanoemulsion
DSE-SNEDDS	*Derris scandens* extract-loaded self-nanoemulsifying drug delivery system
DXA	Dexamethasone
EtOH	Ethanol
FBS	Fetal bovine serum
HPLC	High-performance liquid chromatography
HSP	Hansen solubility parameter
IC_20_	20% inhibitory concentration
IC_50_	Half-maximal inhibitory concentration
LBDDS	Lipid based drug delivery systems
LOD	Limit of detection
LOQ	Limit of quantification
LPS	lipopolysaccharide
MCT	medium-chain triglyceride
MTT	3-(4,5-Dimethylthiazol-2-yl)-2,5-diphenyltetrazolium bromide
ND	not determined
NE	Nanoemulsion
NO	Nitric oxide7
NSAIDs	Non-steroidal anti-inflammatory drugs
PCS	Photon correlation spectroscopy
PDA	Photodiode array detector
PdI	Polydispersity index
PLS	Partial least squares model
Q^2^	Prediction power
R^2^	Goodness of fit
SEM	Standard error of the mean
SMEDDS	Self-microemulsifying drug delivery systems
SNEDDS	Self-nanoemulsifying drug delivery systems
