# Supplementary material for: Design and Optimization of Self-Nanoemulsifying Drug Delivery Systems to Enhance the Anti-Inflammatory Efficacy of Derris scandens (Roxb.) Benth. Extract
Source: Pharmaceutics. 2026 Sep 20;18(9):1188. doi: 10.3390/pharmaceutics18091188 (PMC13610951; doi:10.3390/pharmaceutics18091188)
Supplement: Supplementary file 1 [file pharmaceutics-18-01188-s001.zip › pharmaceutics-4515697-supplementary updated.pdf]

pharmaceutics

MDPI

Supplementary Materials

Design and optimization of self-nanoemulsifying drug delivery systems to enhance the anti-inflammatory efficacy of *Derris scandens* (Roxb.) Benth. Extract

Nattakanwadee Khumpirapang [1], Kantapich Srikham [2], Thapakorn Somboon [3], Krit Suknuntha [4], Daniela Gruľová [5], Neti Waranuch [2,6], Kornkanok Ingkaninan [1,3,*] and Siriporn Okonogi [7,*]

[1] Department of Pharmaceutical Chemistry and Pharmacognosy, Faculty of Pharmaceutical Sciences, Naresuan University, Phitsanulok 65000, Thailand; nattakanwadeek@nu.ac.th
[2] Cosmetics and Natural Products Research Center, Faculty of Pharmaceutical Sciences, Naresuan University, Phitsanulok 65000, Thailand; kantapich.sr@nu.ac.th (K.S.); netiw@nu.ac.th (N.W.)
[3] Center of Excellence for Innovation in Chemistry, Faculty of Pharmaceutical Sciences, Naresuan University, Phitsanulok 65000, Thailand; thapakorn.so@nu.ac.th
[4] Department of Pharmaceutical Chemistry, Faculty of Pharmaceutical Sciences, Prince of Songkla University, Songkhla 90110, Thailand; krit@pharmacy.psu.ac.th
[5] Department of Ecology, Faculty of Humanities and Natural Sciences, University of Prešov, Prešov 08001, Slovakia; daniela.grulova@unipo.sk
[6] Department of Pharmaceutical Technology, Faculty of Pharmaceutical Sciences, Naresuan University, Phitsanulok 65000, Thailand
[7] Center of Excellence in Pharmaceutical Nanotechnology, Faculty of Pharmacy, Chiang Mai University, Chiang Mai 50200, Thailand
* Correspondence: kornkanoki@nu.ac.th (K.I.); siriporn.okonogi@cmu.ac.th (S.O.); Tel.: +66-5596-1860 (K.I.) Tel.: +66-5394-4311 (S.O.)

HPLC Method Validation Data for the Quantification of Lupalbigenin

Linearity and calibration range

The linearity of the detector response was examined using lupalbigenin standard solutions at six concentration levels: 2, 5, 10, 25, 50, and 100 µg/mL. The integrated chromatographic peak area was plotted against the corresponding analyte concentration (Figure S1). A linear response was obtained throughout the evaluated range, with a coefficient of determination (R^2) of 0.9999. The calibration model was therefore considered suitable for the quantitative determination of lupalbigenin between 2 and 100 µg/mL.

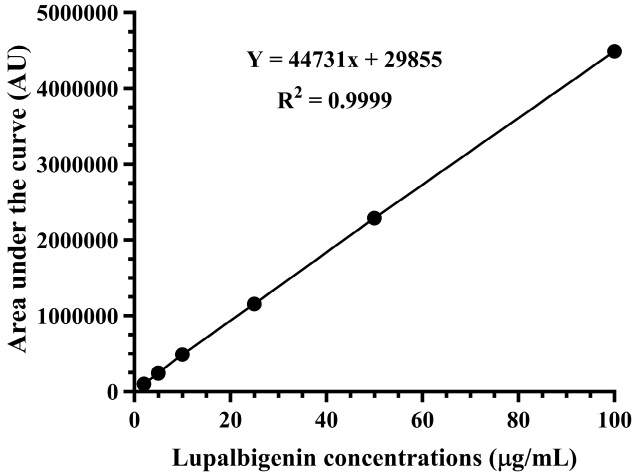

Figure S1. A standard calibration curve demonstrating the correlation between lupalbigenin concentration and its peak area.

Detection and quantification limits

Instrumental sensitivity was examined using low-concentration lupalbigenin standard solutions. The experimentally assigned detection and quantification limits were 0.5 and 2.0 µg/mL, respectively. The signal-to-noise measurements obtained at these concentrations are summarized in Table S1.

Table S1. Signal-to-noise data at the experimentally assigned LOD and LOQ.

Parameter	Concentration (µg/mL)	Mean S/N	SD	SEM	*n*
LOD	0.5	12.59	1.84	1.06	3
LOQ	2.0	54.49	6.43	3.71	3

Note: S = signal and N = noise

Accuracy

Accuracy was evaluated by standard addition at three levels (4, 40, and 70 µg/mL). Each level was analyzed in triplicate. Recovery was calculated from the difference between the lupalbigenin concentration measured in the fortified sample and that measured in the unfortified sample, relative to the measured amount of standard added (Table S2 and S3).

$$Recovery\ (\%) = [(C_{fortified} - C_{unfortified}) / C_{added}] \times 100$$

Table S2. Individual measurements used in the lupalbigenin recovery assessment.

Nominal addition (μg/mL)	Replicate	Fortified sample (μg/mL)	Standard added (μg/mL)	Unfortified sample (μg/mL)	Recovery (%)
4	1	24.49	4.10	20.50	97.38
	2	23.95	4.06	19.87	100.76
	3	23.38	4.02	18.99	109.14
40	1	61.56	41.34	18.05	105.26
	2	61.87	41.39	18.44	104.92
	3	61.89	41.38	18.10	105.83
70	1	94.96	72.63	18.05	105.89
	2	94.92	72.65	18.44	105.27
	3	95.28	72.65	18.10	106.25

Table S3. Summary statistics for the recovery results.

Nominal addition (μg/mL)	*n*	Mean recovery (%)	SD	SEM	RSD (%)
4	3	102.43	6.05	3.50	5.91
40	3	105.34	0.46	0.27	0.44
70	3	105.80	0.50	0.29	0.47
Overall	9	104.52	3.43	1.14	3.29

Across the nine recovery measurements, the mean recovery was 104.52% with an SEM of 1.14%. Recoveries at the individual fortification levels ranged from 97.38% to 109.14%.

Precision

- **Repeatability**

Repeatability was assessed within a single analytical run under the same operating conditions. Lupalbigenin standards at 4, 40, and 70 μg/mL were each analyzed three times. The replicate measurements and statistics calculated from those values are shown in Table S4.

Table S4. Repeatability of the HPLC determination of lupalbigenin.

Nominal (µg/mL)	Rep. 1	Rep. 2	Rep. 3	Mean (µg/mL)	SD	SEM	RSD (%)	Acceptance limit (RSD (%))
4	4.07	4.06	4.01	4.05	0.032	0.019	0.79	6.45
40	41.34	41.39	41.38	41.37	0.026	0.015	0.06	4.57
70	72.63	72.65	72.65	72.64	0.012	0.007	0.02	4.20

- **Intermediate precision**

Intermediate precision was evaluated over three analytical days. On each day, three replicate measurements were obtained at each concentration level, giving nine measurements per level. The daily observations are provided in Table S5, and the corresponding summary statistics are reported in Table S6.

Table S5. Individual measurements collected for the intermediate-precision study.

Nominal concentration (µg/mL)	Day	Replicate 1 (µg/mL)	Replicate 2 (µg/mL)	Replicate 3 (µg/mL)
4	1	4.16	4.04	4.18
	2	4.07	4.06	4.01
	3	4.10	4.06	4.02
40	1	41.92	41.90	41.82
	2	41.34	41.39	41.38
	3	41.59	41.09	41.37
70	1	73.25	73.45	73.52
	2	72.63	72.65	72.65
	3	70.48	70.35	70.11

Table S6. Intermediate-precision statistics calculated across three analytical days.

Nominal concentration (µg/mL)	*n*	Mean (µg/mL)	SD	SEM	RSD (%)	Acceptance limit (RSD (%))
4	9	4.08	0.06	0.02	1.44	12.90
40	9	41.53	0.29	0.10	0.70	9.14
70	9	72.12	1.40	0.47	1.94	8.40

The observed RSD (%) values for both repeatability and intermediate precision were below the acceptance limits reported in the validation dataset at all three concentration levels.
